# *Toxoplasma* GRA Peptide-Specific Serologic Fingerprints Discriminate Among Major Strains Causing Toxoplasmosis

**DOI:** 10.3389/fcimb.2021.621738

**Published:** 2021-02-19

**Authors:** David Arranz-Solís, Cristina G. Carvalheiro, Elizabeth R. Zhang, Michael E. Grigg, Jeroen P. J. Saeij

**Affiliations:** ^1^Pathology, Microbiology and Immunology Department, Veterinary Medicine School 3A, University of California Davis, Davis, CA, United States; ^2^Laboratory of Parasitic Diseases, Molecular Parasitology Section, National Institute of Allergy and Infectious Diseases, National Institutes of Health, Bethesda, MD, United States; ^3^Department of Pediatrics, Ribeirão Preto Medical School, University of São Paulo, Ribeirão Preto, Brazil

**Keywords:** *Toxoplasma*, serotyping, peptide, ELISA, dense granule, rhoptry

## Abstract

The severity of toxoplasmosis depends on a combination of host and parasite factors. Among them, the *Toxoplasma* strain causing the infection is an important determinant of the disease outcome. Type 2 strains dominate in Europe, whereas in North America type 2, followed by type 3 and 12 strains are commonly isolated from wildlife and patients. To identify the strain type a person is infected with, serological typing provides a promising alternative to the often risky and not always possible biopsy-based DNA methods of genotyping. However, despite recent advances in serotyping, improvements in the sensitivity and specificity are still needed, and it does not yet discriminate among the major *Toxoplasma* lineages infecting people. Moreover, since infections caused by non-1/2/3 strains have been associated with more severe disease, the ability to identify these is critical. In the present study we investigated the diagnostic potential of an ELISA-based assay using 28 immunogenic *Toxoplasma* peptides derived from a recent large-scale peptide array screen. Our results show that a discrete number of peptides, derived from *Toxoplasma* dense granule proteins (GRA3, GRA5, GRA6, and GRA7) was sufficient to discriminate among archetypal strains that infect mice and humans. The assay specifically relies on ratios that compare individual serum reactivities against GRA-specific polymorphic peptide variants in order to determine a “reactivity fingerprint” for each of the major strains. Importantly, nonarchetypal strains that possess a unique combination of alleles, different from types 1/2/3, showed either a non-reactive, or different combinatorial, mixed serum reactivity signature that was diagnostic in its own right, and that can be used to identify these strains. Of note, we identified a distinct “HG11/12” reactivity pattern using the GRA6 peptides that is able to distinguish HG11/12 from archetypal North American/European strain infections.

## Introduction

The intracellular protozoan parasite *Toxoplasma gondii* is the causative agent of toxoplasmosis in humans and animals. Infection usually occurs after ingestion of tissue cysts present in undercooked meat or by ingestion of soil, water or food contaminated with oocysts shed in feline feces (Robert-Gangneux and Dardé, 2012). In humans, the worldwide seroprevalence is estimated to be approximately 30%, although it can vary widely (10–80%) between countries (Robert-Gangneux and Dardé, 2012; Dard et al., 2016). Although infection is usually benign with occasional flu-like symptoms in immunocompetent individuals, it can also cause severe disease such as ocular toxoplasmosis or encephalitis, especially in immunocompromised patients, as well as abortion or birth defects in pregnant women infected for the first time (Furtado et al., 2011). Hence, progression and severity of toxoplasmosis depends on a combination of host and parasite factors, with the infecting *Toxoplasma* strain type playing a pivotal role in the outcome of disease (Boothroyd and Grigg, 2002; Ajzenberg et al., 2010; Fekkar et al., 2011).

*Toxoplasma* has a markedly clonal population structure in North America and Europe, with four distinct clonotypes commonly isolated from animals and humans (types 1, 2, 3 and 12), although type 2 strains predominate, especially in Europe (Dubey et al., 2011; Herrmann et al., 2012; Su et al., 2012). By contrast, the majority of characterized isolates from South America are genetically distinct from the North-American/European strains and have been referred to as non-canonical, non-archetypal, non-clonal or atypical strains or lineages (Grigg and Sundar, 2009; Su et al., 2012). Some of these have been associated with severe systemic toxoplasmosis in immunocompetent people (Carme et al., 2009; Hamilton et al., 2019). Conversely, type 2 strains are less often associated with severe clinical disease. In fact, type 1 and non-type 2 strains are more likely to be found infecting immunocompetent individuals suffering from ocular toxoplasmosis and are disproportionately associated with severe congenital toxoplasmosis in the USA and Europe (Howe et al., 1997; Fuentes et al., 2001; Grigg et al., 2001; Carme et al., 2002; Nowakowska et al., 2006; Carme et al., 2009; McLeod et al., 2012; Shobab et al., 2013; Hutson et al., 2015). In addition, the “Cougar” strain, which was isolated from oocysts shed by mountain lions (*Puma concolor*) and belongs to haplogroup 11 (HG11) (Su et al., 2012; Lorenzi et al., 2016), has been epidemiologically linked to a major waterborne toxoplasmosis outbreak in Victoria, British Columbia, Canada (Aramini et al., 1998; Aramini et al., 1999). Similarly, a recent outbreak in Wisconsin, USA, was reported to be caused by consumption of undercooked venison contaminated with cysts from a HG12 strain (Schumacher et al., 2020). It is worth mentioning that in both of these outbreaks, infected patients experienced systemic disease, suggesting that either the number of cysts/oocysts ingested was exceptionally high or that a virulent strain was involved.

Recent advances in genotyping *Toxoplasma* isolates from different continents have identified extensive genetic diversity; however, genotyping has substantial limitations: it is both time consuming and costly, and it often requires parasite isolation by invasive and risky biopsies, which is normally only achieved in severe clinical cases (Robert-Gangneux and Dardé, 2012; Su et al., 2012; Shobab et al., 2013; Lorenzi et al., 2016). In contrast to DNA-dependent techniques, serological typing allows for the inclusion of clinical, as well as subclinical, cases, and represents a rapid, sensitive, and relatively non-invasive alternative (Kong et al., 2003). Serotyping takes advantage of the fact that *Toxoplasma* stimulates a strong and persistent humoral immune response, eliciting the production of antibodies against parasite proteins that are often highly polymorphic among distinct strains. By using polymorphic peptides as antigens and monitoring the reactivity of the serum, a prediction of the infecting strain type can be made. Indeed, several studies employed synthetic peptides or recombinant polypeptides derived from polymorphic regions of *Toxoplasma* secreted/excreted proteins to serologically distinguish type 2 from non-type 2 infections in a variety of hosts (Kong et al., 2003; Peyron et al., 2006; Morisset et al., 2008; Xiao et al., 2009; Sousa et al., 2009; Sousa et al., 2010; Vaudaux et al., 2010; Maksimov et al., 2012b; Maksimov et al., 2012a; McLeod et al., 2012; Shobab et al., 2013; Hutson et al., 2015; Maksimov et al., 2018). Other studies have attempted to develop peptides capable of differentiating type 1 *vs.* 3 and type 2 *vs.* 3 infections, but with only partial success (Xiao et al., 2009; Maksimov et al., 2012a; Maksimov et al., 2018). We recently developed a discrete set of *Toxoplasma* polymorphic peptides capable of discriminating infections caused by type 1, 2 and 3 strains (Arranz-Solís et al., 2019), but these have not yet been widely applied, nor have they been systematically tested against serum collected from patients infected with non-archetypal strains.

Although the serotyping approach lacks the exquisite sensitivity and specificity of DNA genotyping, the recent availability of whole genome sequences from 64 different *Toxoplasma* strains, including atypical ones (Lorenzi et al., 2016), makes it possible to test whether serologic reactivity is determined by the epitope present, and not the underlying genotype of the parasite strain. Based on our previous work, we observed that many atypical strains either possess a type 1/2/3 epitope within the GRA peptides, even though the allele present is not type 1/2/3, or they share the sequence of one of the type 1, 2 and/or 3 strains but in different admixture combinations (Lorenzi et al., 2016). Since arrays are not always readily available and entail a great cost, the aim of the present study was to test a small selection of promising peptides using a larger collection of murine and human serum samples by ELISA, which represents an easier, more accessible and affordable assay platform that is available in most laboratories. To this end, a total of 28 *Toxoplasma* allele-specific peptides were designed and synthesized for screening. By using a ratio that compares the reaction of different variants within the same polymorphic regions, an approach taken by McLeod et al. (2012), we demonstrate that peptides derived from the GRA proteins GRA3, GRA5, GRA6, GRA7 and GRA15 differentiate between types 1, 2, 3, as well as other strain infections produced by non-archetypal strains. These peptides should enable further research to assess whether the genotype of the infecting strain is associated with specific disease phenotypes among different populations, allowing for a better understanding of the molecular epidemiology of toxoplasmosis.

## Methods

### *Toxoplasma In Vitro* Culture

*Toxoplasma* strains used for animal infections and lysate production (see below) were routinely maintained on Human Foreskin Fibroblast (HFF) monolayers as previously described (Jensen et al., 2015).

### Ethics Statement and Mice Infection

All animal experiments were performed in strict accordance with the recommendations in the Guide for the Care and Use of Laboratory Animals of the National Institutes of Health. The MIT Committee on Animal Care (assurance number A-3125-01), the Institutional Animal Care and Use Committee (IACUC) at the University of California, Davis (assurance number A-3433-01), and the Animal Care and Use Committee (ACUC) of the Intramural Research Program of NIAID (Animal study protocol; LPD22E) approved all protocols, and all efforts were made to minimize unnecessary distress to the animals. The animal study protocol LPD22E was reviewed and approved by the Animal Care and Use Committee of the Intramural Research Program of the National Institute of Allergy and Infectious Diseases (NIAID) at the National Institutes of Health.

### Mouse Serum Samples

A summary of the mouse breed, parasite, dose, route, treatment and time of collection of sera is presented in **Supplemental File 1A**. Serum samples from mice infected with RH (type 1); M4, ME49 and PRU (type 2); CEP and CL14 (type 3); MAS (type 4), BOF (type 6), CAST (type 7), P89 (type 9), GUY-DOS (type 10), and Cougar (type 11) were used (Jensen et al., 2015; Mukhopadhyay et al., 2020, This study). In addition, samples from mice infected orally with cysts from the type 2 PRU and type 3 CEP strains, as well as intraperitoneally with oocysts of the type 2 M4 strain, were also included. Finally, the most reactive peptides were tested with a set of sera from mice infected with the strains S22, S26, CL13, and STE10, which are F1 progeny from a cross between type 2 (ME49) and 3 (CEP) strains in cats (Sibley et al., 1992). Animals were checked every day to detect signs of illness, such as rough hair coat, apathy or weight loss. Depending on the strain, sulfadiazine, alone or in combination with pyrimethamine, was added to the drinking water (0.4 mg/ml and 0.2 mg/ml, respectively), either at the time of infection or upon detection of clinical signs, to control the acute stage. The sera of infected mice were collected at different time points according to the severity of infection caused by specific strains. Serum samples from individual mice were pooled for each group of infection and used for the ELISAs.

### Human Serum Samples

Thirty-four serum samples from infected patients from France, Brazil or the United States were used in the present study (de-la-Torre et al., 2013; Arranz-Solís et al., 2019; This study) (**Supplemental File 1B**). The strain causing the infection was previously assessed by genotyping or serotyping employing GRA6 and GRA7 peptides (6I/III, d6I/III, 6II and 7II) originally described by Kong et al. (2003). Samples included infections caused by type 1, 2, 3, 1/3 and SA (aka atypical) strains. In addition, a number of samples were defined as non-reactive, or NR (i.e., positive serum reactivity against either *Toxoplasma* lysate or rSAG1 antigen with no reaction to the Kong panel of peptides). These patient samples are thought to represent infection by atypical strains (de-la-Torre et al., 2013; Shobab et al., 2013). Human samples were used according to the Committee on the Use of Humans as Experimental Subjects (COUHES) application No. 0808002869.

### *Toxoplasma* Lysate Production

A *Toxoplasma* soluble lysate extract was prepared to be used in the ELISA experiments. To achieve a homogeneous representation of the type 1, 2, and 3 strains, which generally encompass most peptide polymorphisms, *Toxoplasma* tachyzoites of RH and GT1 (type 1); PRU, ME49, and M4 (type 2); and VEG and CEP (type 3) strains were grown as described above. When parasites started to lyse out, flasks were scraped and tachyzoites recovered and syringe lysed (27×G and 30×G). Pellets were collected by centrifugation at 700×g for 7 min and washed twice with 1×PBS to remove residual culture media. Washed pellets were then resuspended in 5 ml 1×PBS and filtered to remove cells and debris by passing the suspensions through PD-10 desalting columns (GE Healthcare). Tachyzoites were counted using a Neubauer chamber and pellets collected by centrifugation and stored at −80 °C until processing. A total of 5×10^9^ tachyzoites were used for the soluble antigen production. For this, pellets were thawed on ice and resuspended thoroughly in a lysate solution containing 10mM Tris, 57 mM phenylmethylsulphonyl fluoride (PMSF), and 1× protease inhibitor + phosphatase cocktail (ThermoFisher) in 1×PBS. All lysates were then mixed and subjected to 3 cycles of 3 min sonication (50% amp 15 s pulse + 10 s pause) (EpiShear Q120AM probe sonicator, Active Motif). To avoid overheating of the samples, tubes were placed on a freeze-cold cooler throughout the process. Finally, sonicated lysates were centrifuged at 14,000×g for 30 min at 4 °C and supernatants containing the soluble antigen transferred to new tubes. Protein concentration was determined by the Bradford method (Bradford, 1976) and aliquots were stored at −80 °C until used.

### Selection, Design and Production of Peptides

In a recent work from our group, we tested 950 peptides from 62 *Toxoplasma* genes by peptide array (Arranz-Solís et al., 2019). A number of promising peptides were found to be immunogenic and strain-specific; however, arrays are not readily available and are often cost prohibitive. Hence, we prioritized a small selection of promising peptides from the array work to develop an ELISA format assay to test against a broader panel of murine and human serum samples. Only antigenic peptides that consistently discriminated the strain of infection were re-synthesized for the ELISA assay. Some selected peptides were shortened from the originally described peptide (Arranz-Solís et al., 2019) to evaluate their capacity to maintain reactivity and the importance of specific amino acids. Altogether, a total of 28 allele-specific peptides from *Toxoplasma* dense granule (GRA3, GRA5, GRA6, GRA7 and GRA15) and rhoptry (ROP8 and ROP20) proteins, as well as one histidine acid phosphatase superfamily protein (TGME_308950) and a PA14 domain-containing protein (TGME49_258400, only expressed in oocysts) were designed (**Table 1**). When not present, cysteine residues were added to the N or C terminus of the peptide for coupling purposes. Peptides were synthesized with a purity of >95% and coupled to Keyhole-Limpet Hemocyanin (KLH) carrier protein (Biomatik USA, Wilmington, DE). In addition, a KLH-coupled control peptide derived from a randomly generated sequence of GRA6 (Kong et al., 2003) was used to rule out nonspecific reactions against KLH. Lyophilized peptides were resuspended in MilliQ water or in low concentrations (0.1–1%) of glacial acetic acid, depending on their hydrophilic properties, to a final concentration of 1 μg/μl, aliquoted and stored at -80 °C until use. All but one peptide were successfully resuspended; unfortunately, all attempts to resuspend the peptide GRA5-III-38 failed, even with increasing concentrations of Dimethyl Sulfoxide (DMSO), acetonitrile or acetic acid. Therefore, this peptide was subsequently excluded from the study.

**Table 1:** List of peptides used in the ELISA test.

Gene ID	Name^a^	Sequence^b^	type	Reference
TGME49_227280	GRA3-I/III-43	ADQP**E**NHQALAEC	1/3	Arranz-Solís et al., 2019 ^c^
TGME49_227280	GRA3-II-43	ADQP**G**NHQALAEC	2	Arranz-Solís et al., 2019 ^c^
TGME49_286450	GRA5-I-38	CSEGA**R**G**R**EQ	1	Arranz-Solís et al., 2019
TGME49_286450	GRA5-II-38	CSEGA**W**G**G**EQ	2	Arranz-Solís et al., 2019
TGME49_286450	GRA5-III-38*	CSEGA**G**G**R**E**R**	3	Arranz-Solís et al., 2019
TGME49_275440	GRA6-I-44	ADS**G**GV**K**QTPC	1	Arranz-Solís et al., 2019 ^c^
TGME49_275440	GRA6-II-44	ADS**G**GV**R**QTPC	2	Arranz-Solís et al., 2019 ^c^
TGME49_275440	GRA6-III-44	ADS**D**GV**K**QTPC	3	Arranz-Solís et al., 2019 ^c^
TGME49_275440	GRA6-II-200	C**N**EGRG**E**GG**EDD**	2	Arranz-Solís et al., 2019
TGME49_275440	GRA6-III-201	CEGRG**Y**GG**RGE**	3	Arranz-Solís et al., 2019 ^c^
TGME49_275440	GRA6-I/III-213	CLHP**ER**VN**V**FD**Y**	1/2	Kong et al., 2003 [6-I/III]
TGME49_275440	GRA6-II-214	CLHP**GS**VN**E**FD**F**	2	Kong et al., 2003 [6-II]
TGME49_275440	GRA6-II-215	CHP**GS**VN**E**FD	2	Kong et al., 2003 ^c^
TGME49_203310	GRA7-I-164	CLTE**E**QQ**R**GDEP	1	Arranz-Solís et al., 2019 ^c^
TGME49_203310	GRA7-III-164	CLTE**Q**QQ**T**GDEP	3	Arranz-Solís et al., 2019 ^c^
TGME49_203310	GRA7-II-224	CVPESG**K**D**G**EDARQ	2	Kong et al., 2003 [7-II]
TGME49_203310	GRA7-III-224	CVPESG**E**D**R**EDA	3	Kong et al., 2003 [(d)7-III]
TGME49_203310	GRA7-II-226	CESG**K**D**G**EDAR	2	Arranz-Solís et al., 2019
TGME49_203310	GRA7-III-226	CESG**E**D**R**EDA	3	Arranz-Solís et al., 2019 ^c^
TGME49_275470	GRA15-II-477	CSPHTLLSRS	2	Arranz-Solís et al., 2019
TGME49_215775	ROP8-I/III-61	CPGGASPRHFHS	1/3	Arranz-Solís et al., 2019
TGME49_215775	ROP8-I/III-305	CSN**T**IKQMK**Q**EV	1/3	Arranz-Solís et al., 2019 ^c^
TGME49_215775	ROP8-II-305	CSN**A**IKQMK**E**EV	2	Arranz-Solís et al., 2019
TGME49_258230	ROP20-I-331	CLRKQG**GN**SLLN	1	Arranz-Solís et al., 2019
TGME49_258230	ROP20-II/III-331	CLRKQG**DT**SLLN	2/3	Arranz-Solís et al., 2019
TGME49_308950	hp-I/II-47	S**IW**RPQGTPEC	1/2	Arranz-Solís et al., 2019 ^c^
TGME49_308950	hp-III-47	S**TL**RPQGTPEC	3	Arranz-Solís et al., 2019 ^c^
TGME49_258400	PA14-I/II/III-35	EFRQQHRKTIDGRLC	1/2/3	This study
CONTROL	KLH1	CEVVHDYRLFNP		Kong et al., 2003

*All attempts to dissolve GRA5-III-38 were unsuccessful.

a I, II, and III refer to the archetypal strain that includes the peptide sequence, and the numbers following these indicate position in the coding sequence of the first amino acid of each peptide. The cysteine (C) at the N or C terminus was added for coupling purposes and was not present in the protein. Hp stands for hypothetical protein. TGME49_308950 is annotated as histidine acid phosphatase superfamily protein.

b Bold type indicates polymorphic sites.

c Modified version of a peptide described in Arranz-Solís et al., 2019 or Kong et al., 2003.

### Mouse ELISA

An indirect enzyme-linked immunosorbent assay (ELISA) was used to determine the reactivity level shown by different serum samples from mice infected with several *Toxoplasma* strains (**Supplemental File 1A**). Peptides (**Table 1**) were used to coat 96-well microtiter plates (CLS3590, Corning, Sigma). For this, 100 μl/well of peptides at 10 μg/ml (1 μg/well) diluted in carbonate buffer (100 mM, pH 9.6) were incubated overnight at 4 °C. For each sample, the *Toxoplasma* lysate (0.25 μg/well) and the KLH-coupled control peptide were used as positive and negative controls, respectively. Subsequently, non-specific binding was blocked by adding 200 μl of bovine serum albumin diluted to 3% in PBS (pH 7.4) containing 0.05% Tween 20 (PBS-T). After 2 h incubation at room temperature (RT), plates were washed four times with PBS-T. Subsequently, 100 μl of 1/100 sera dilution in block solution was added to each well and incubated for 1 h at 37 °C. In each plate, samples of the same positive and negative control sera were included. These corresponded to a pool of mice infected with the M4 strain and uninfected mice, respectively. After four washes in PBS-T, 100 μl of horseradish peroxidase (HRP) conjugate-labeled goat α-mice IgG antibody (16-078, Thermo-Fisher Scientific) diluted 1:2,000 in PBS-T was added and incubated for 1 h at 37 °C. Plates were washed as above before the addition of 100 μl per well of ABTS substrate (11684302001, Sigma) and incubated at room temperate in the dark. The reaction was stopped by the addition of 100 μl per well of a solution of 0.3 M oxalic acid, and the optical density (OD) was read at 405 nm (OD_405_) in a plate reader machine (Molecular Device Spectramax M2e). Plates were stopped when the positive control (lysate) reached OD values of 1.5–1.8, usually within 15–30 min. To normalize the obtained OD values, for each sample and peptide a relative index percent (RIPC) was calculated using the following formula RIPC = (peptide OD_405_ sample – peptide OD_405_ negative control)/(lysate OD_405_ sample – lysate OD_405_ negative control) × 100 (**Supplemental File 2**). To use a more stringent normalization, we decided to employ constant values for both the peptides and lysate OD negative controls. These values were calculated as two times the average of the ODs obtained with the negative control for each peptide and the lysate in all the plates used (0.39 and 0.25, respectively). Since each peptide showed a different level of reactivity, we decided to calculate a ratio between those homologous peptides from the different strain types (for example, GRA3-I/III-43 *vs.* GRA3-II-43). To further normalize the results, before calculating the ratios, RIPC values below 1 (including negative values) were converted to 1, and those higher than 100 were converted to 100.

### Human ELISA

Human ELISA assays were performed at the Laboratory of Parasitic Diseases, Bethesda, MD, USA, as described above for mice with the following modifications: 50 μl per well were used in all steps using a different set of 96-well plates (353912, BD Falcon). Peptides were provided by the Saeij lab and labelled with letter codes to ensure that all ELISAs were performed blinded and an unbiased interpretation of results was carried out. To validate the reproducibility of our test and the status of the serum samples, three control peptides, 6I/III, 6II and 7II (Kong et al., 2003), well-known to be able to differentiate type 2 *vs.* non-type 2 infections, were included. These peptides shared the same sequence as our GRA6-I/III-213, GRA6-II-214 and GRA7-II-224 (**Table 1**), although they were manufactured by a different provider and manually coupled with the KLH carrier protein (Shobab et al., 2013). In addition, for each sample a positive control consisting of a universal and highly reactive recombinant SAG1 (rSAG1) antigen was included (Shobab et al., 2013). As a blocking buffer, a 2% casein (C5890, Sigma) solution in PBS was used, whereas several sample dilutions (1/40-1/160) were done for each patient and incubated at room temperature for 1 h. All coupled peptides were reacted with a cohort of 30 negative sera and each serum was tested against an irrelevant peptide-KLH coupled control. As a secondary antibody, a 1/1,000 dilution of the HRP Anti-Human IgG HRP (555788, BD Pharmingen) was used. Finally, after addition of the ABTS reagent, OD_405_ values were obtained for each sample by reading the plate at different time points ranging from 20–40 min for the rSAG1 antigen to 2–4 h for the peptides. Data were presented as an OD index, normalized as described previously (Kong et al., 2003; Shobab et al., 2013). Briefly, the OD_405_ value for each serotyping peptide was divided by the OD_405_ reading for the irrelevant peptide-KLH control and the results expressed as arbitrary units. Serum samples from individuals infected with *T. gondii* for whom the genotype is known served as positive controls for each experiment. Thus, it is expected that samples unreactive to specific peptides have a normalized value close to 1. Threshold values above which each sample-peptide reaction is considered positive were determined by adding 2 standard deviations (SDs) to the average of the normalized OD ratio derived from the 30 seronegative samples. Values were normalized by dividing homologous peptides from the different strain types as described above for the mice ELISA.

## Results

### Peptide ELISA Using Sera From *Toxoplasma* Infected Mice

**Figure 1:**
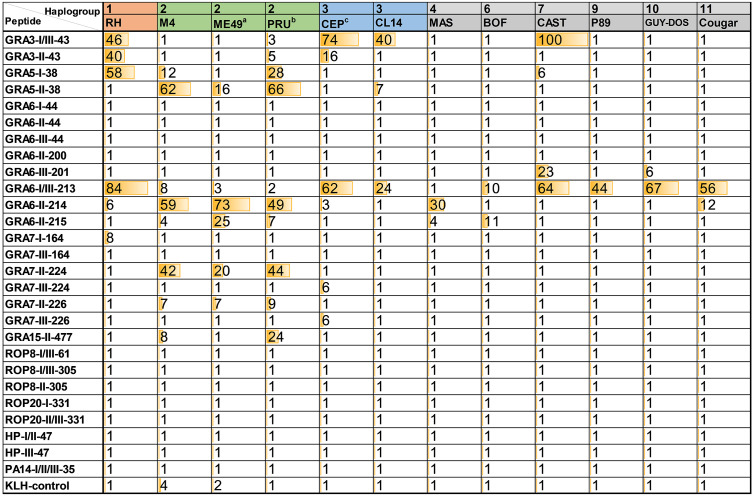
Relative index percentage (RIPC) values for ELISAs using *Toxoplasma* peptides and murine sera. OD values for each sample and peptide were normalized by calculating a relative index percentage (RIPC) with the following formula RIPC = (peptide OD_405_ sample – peptide OD_405_ negative control)/(lysate OD_405_ sample – lysate OD_405_ negative control) × 100. To use a more stringent normalization, constant OD values corresponding to two times the average of the ODs obtained with the negative sample for all peptides and the lysate (0.39 and 0.25, respectively) were used. In addition, RIPC values below 1 (including negative values) were converted to 1, and those higher than 100 were converted to 100. ^a^ average of 2 ME49 samples; ^b^ average of 7 PRU samples; ^c^ average of 6 CEP samples.

Sera from mice infected with different *Toxoplasma* strains were used to test the antigenicity of the peptides described in **Table 1**. Namely, sera from mice infected with one type 1 (RH) strain, three type 2 (M4, ME49, PRU), two type 3 (CEP, CL14), one MAS (type 4), BOF (type 6), CAST (7), P89 (9), GUY-DOS (10) and Cougar (11) strains were assessed to detect specific reactivity. As expected, all these sera showed a strong reactivity against the *Toxoplasma* lysate (soluble antigen), whereas the uninfected negative serum sample did not react against any of the peptides nor the lysate (**Supplemental File 2****)**. In addition, none but two showed reactivity against the KLH-control peptide (RIPC of 4 and 7 for one ME49 and one M4 sample, respectively), confirming the absence of (or very low) nonspecific reactivity of the samples to the KLH carrier protein. From the final 27 peptides that were tested by ELISA, 14 (GRA3-I/III-43, GRA3-II-43, GRA5-I-38, GRA5-II-38, GRA6-III-201, GRA6-I/III-213, GRA6-II-214, GRA6-II-215, GRA7-I-164, GRA7-II-224, GRA7-III-224, GRA7-II-226, GRA7-III-226 and GRA15-II-477) showed reactivity with at least one of the examined serum (**Figure 1**). None of the other 13 peptides showed reactivity with any of the samples and were therefore not further studied.

The reactivity of different serum samples obtained from mice infected with the same strain but that differed in mouse breed, infection dose, treatment, route of infection or day of collection (**Supplemental File 1A**) was investigated with these 14 peptides. Although a clear pattern was not found when comparing samples from the strains ME49 (type 2, 2 samples), PRU (type 2, 7 samples) and CEP (type 3, 6 samples), this allowed us to confirm the reproducibility of our assay. To simplify the results, an average of the different samples tested for each of the type 2 and 3 strains was calculated (**Figure 1**). The individual values for each sample, as well as the standard deviation of type 2 and 3 strains is shown in **Supplemental File 3**.

### Sera of Mice Infected With Different *Toxoplasma* Strains React to GRA Peptides in an Epitope-Specific Manner

**Figure 2:**
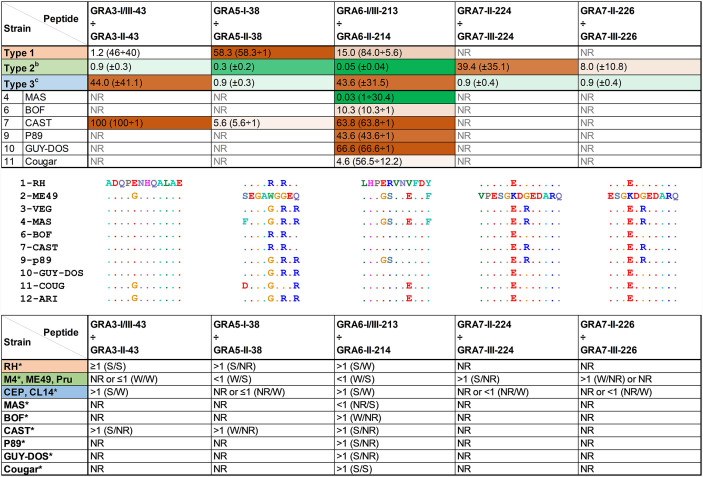
Different *Toxoplasma* strains show a unique profile of seroreactivity against specific GRA peptides. Ratio values from strain-specific peptides assayed with serum samples from mice infected with different *Toxoplasma* strains. Ratios were calculated by dividing the RIPC values of specific peptides with the homologous version from the different strain types (in parenthesis). Ratios greater than 1 were highlighted with an increasing brown color (being 100 the most intense), whereas values between 1 and 0 were highlighted in an increasing green color (being 0 the most intense). NR, non-reactive, designates ratios of 1 derived from samples that did not react to any of the two peptides. As a reference, type 1 RH, as well as the average ratio for individual M4, ME49 and PRU type 2 (^b^) and CEP and CL14 type 3 (^c^) samples are shown. Readers are referred to **Supplemental File 3** for details on individual values and standard deviation. The specific peptide amino acid sequence for each of the strains is displayed in the middle of the figure, where dots represent identical residues. Type 12 Ari strain has been added for comparative purposes. At the bottom, predictions were made based on the ratio obtained for each infection type. Ratios were classified as non-reactive (NR, when both peptides in the division had an RIPC value of 1), lower than 1 (<1), lower but close or almost equal to 1 (≤1), higher but close or almost equal to 1 (≥1) or higher than 1 (>1). The strength (S, strong; W, weak) of each peptide is indicated in parenthesis and separated by a division mark (/). * indicates a single strain result.

Peptides that showed good reactivity were derived from GRA proteins: GRA3, 5, 6, 7 and 15. Among these, GRA3-I/III-43, GRA5-II-38, GRA6-I/III-213, GRA6-II-214 and GRA7-II-224 showed a strong and specific reactivity against their respective type. On the other hand, although GRA6-II-215, GRA7-I-164, GRA7-III-224, GRA7-III-226 and GRA15-II-477 were also very strain specific, their response was weak and not always present in all samples from their respective type (**Figure 1**, **Supplemental File 3**). Nevertheless, while an absence of reactivity is the most likely scenario for these latter peptides, provided a reaction is detected, they are capable of predicting the strain causing the infection (potentially for all three type 1, 2 and 3 strains) in a very specific way. The C-terminal part of GRA6 is a very antigenic region that has been previously shown to represent an excellent epitope for serotyping (e.g. Kong et al., 2003; Arranz-Solís et al., 2019). In this work, we observed that GRA6-II-214 (LHPGSVNEFDF) shows a high and specific reactivity against type 2 serum samples, while it dramatically decreases to almost basal levels in GRA6-II-215 (HPGSVNEFD), which lacks the first and last amino acids compared to GRA6-II-214. Moreover, the type 1/3 version of GRA6-II-214 (GRA6-I/III-213) showed an interesting pattern in which all non-type 2 sera reacted to a certain degree to this peptide (except the type 4 MAS, which has the type II sequence), with the lowest reactivity being observed in few type 2 strains and type 6 BOF (**Figure 1** and **Supplemental File 3**). Except for the latter, the constant amino acid in all the strains reacting to this peptide is the last one (F/Y). Only those sharing the type 1/3 (Y) showed reactivity, even when they possess one or two different amino acids in the rest of the peptide (**Figure 2**). However, the amino acid in position 221 (E/V) also seems to influence the reactivity, as apart from the type 2 strains and type 4 MAS (that share the same sequence), only Cougar has reactivity against the type 2 version of this peptide (GRA6-II-214). Remarkably, while all the samples reacted strongly to either one of the two versions of this portion of GRA6 (GRA6-I/III-213 or GRA6-II-214), type 11 Cougar reacted to both, although with a stronger response to the type 1/3 version. The importance that single amino acid substitutions can have on peptide recognition was also observed in GRA7-224/226 and GRA3-43 peptides (K/E and G/E, respectively).

### A Ratio Between the Seroreactivity Against Different Strain Versions of the Same Peptide Provides a Strain-Specific Signature

Although most sera reacted better to peptide versions corresponding to the infecting strain, they also did to a lesser extent to other versions of the peptide. Hence, we decided to further analyze the RIPC values for each group of related peptides by calculating a ratio of reactivity against different versions of the peptide (**Figure 2** and **Supplemental File 3**). From these ratios it was clear that a distinction between type 1, 2 and 3 strains is possible by using the aforementioned GRA peptides in combination. For example, RIPC values from GRA3 and GRA5 peptides showed a high, often nonspecific, reactivity. However, when RIPC values for GRA3-I/III-43 *vs.* GRA3-II-43 and GRA5-I-38 *vs.* GRA5-II-38 were analyzed, a clear specific ratio was detected for their respective type (**Supplemental File 3**). Moreover, the GRA3-I/III-43 *vs.* GRA3-II-43 ratio proved better for differentiating type 3 *vs.* non-type 3 strains (>1 *vs.* ~1 or non-reactive), whereas the GRA5-I-38 *vs.* GRA5-II-38 ratio was more useful to differentiate type 1 *vs.* non-type 1 strains (>1 *vs.* ≤1 or non-reactive). Apart from the GRA6-I/III-213 *vs.* GRA6-II-214 combination that has already been described to distinguish type 2 *vs.* non-type 2 strains and that we have confirmed here, ratio values from the GRA7 peptides also proved useful to discriminate between type 2 and non-type 2, and occasionally type 3, strains. Nevertheless, although all 4 peptides were very specific, the GRA7-II-224 and GRA7-III-224 showed a stronger reactivity compared to their shorter versions (GRA7-II-226 and GRA7-III-226).

Furthermore, the non-archetypal strains tested in the present study behaved in a way compatible with a mixture of types 1, 2, and/or 3 “standard” profiles. For example, CAST (type 7) reacted as a type 3 strain for the GRA3, GRA6 and GRA7 peptides, while it induced a type 1-like reaction against the GRA5 peptides. In all these cases CAST shares the respective type 3 and 1 sequences (**Figure 2**). On the other hand, Cougar (type 11) showed a unique mixed pattern of type 2 and 1/3 reactivity against only two peptides (GRA6-I/III-213 and GRA6-II-214). The Cougar type 11 strain has a distinct GRA6 allele that shares both type I/III and type II characteristics, likely explaining its low GRA6-I/III-213 *vs.* GRA6-II-214 ratio (4.6) when compared to the other strains with ratios >1 that usually were higher than 10 (**Figure 2**). This represents a unique pattern for these GRA6 peptides that is not observed for any other strain tested here (**Supplemental File 3**). Therefore, this pattern observed with Cougar, in which among all peptides it only reacted against this GRA6 pair, could be diagnostic of this strain. Previously, a similar mixed reactivity against these two peptides was observed for other non-type 1/2/3 strains belonging to the haplogroup (HG)12, such as Ari or Ray (McLeod et al., 2012). Since these strains share with Cougar the same peptide sequence (LHPERVNEFDY) for this portion of GRA6 (**Figure 2**), this pattern could potentially be used to differentiate type 11/12 strains from other strains (see below). Interestingly, apart from CAST, all the other non-archetypal strains tested in the present work only reacted to some of the GRA6 peptides. BOF (type 6) showed a similar low reactivity against GRA6-I/III-213 and GRA6-II-215, but did not react against GRA6-II-214, as opposed to the other strains which showed a stronger reactivity for GRA6-II-214 compared to GRA6-II-215. On the other hand, P89 (type 9) only reacted to GRA6-I/III-213. Taken together, this combination of peptide ratios revealed a unique profile for each of the strains tested that can be potentially used to define a fingerprint for identification, except for GUY-DOS and P89 that showed a very similar signature (**Figure 2**).

### Recombinant and Non-Archetypal Strains Display Different Patterns

**Figure 3:**
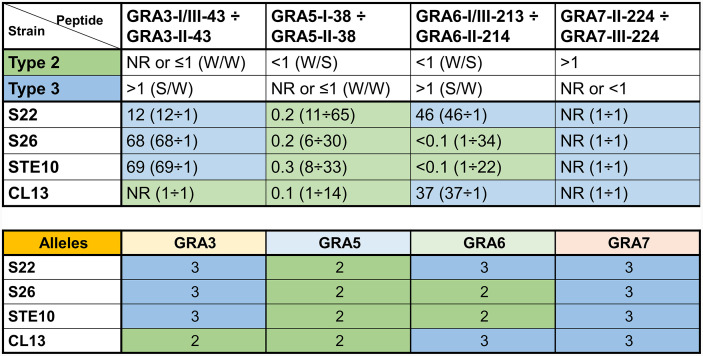
Strains derived from a 2×3 cross can be identified by seroreactivity against a combination of different GRA peptides. Ratio values of reactivity from strain-specific peptides assayed with serum samples from mice infected with *Toxoplasma* S22, S26, STE10 and CL13 strains from a 2×3 cross (Sibley et al., 1992). Ratios were calculated by dividing the RIPC values (in parenthesis) of specific peptides with their homologous peptide from the different strain types. Numbers above 1 were rounded to decrease decimals (readers are referred to **Supplemental File 3** for the exact values). NR, non-reactive, designates ratios of 1 derived from samples that did not react to any of the two peptides. At the top, the observed behavior of type 2 (M4, ME49 and PRU) and 3 (CEP and CL14) samples are shown as a reference (**Figure 2**), where the strength (S, strong; W, weak) of each peptide is indicated in parenthesis and separated by a division mark (/). Predictions were made based on this and highlighted in green and blue for type 2 and 3 alleles, respectively. At the bottom, the actual alleles for each strain that can be found at http://toxomap.wustl.edu/IIxIII_Typing_Table.html are shown.

Since a low reactivity against few peptides was the pattern observed in most of the non-archetypal strains, but not in any of the type 1, 2 or 3 strains, we next investigated whether non-archetypal strains could be differentiated from recombinant strains derived from type 2×3 crosses. To this end, the GRA3, GRA5, GRA6 and GRA7 peptides were tested in combination for their ability to determine the allele type by using sera from mice infected with four different strains derived from a 2×3 cross (Sibley et al., 1992): S22, S26, STE10 and CL13. A prediction of the type for each of these proteins at the GRA3, GRA5 and GRA6 loci was made based on the RIPC ratios (**Figure 3**), which was further confirmed with the genotype of these F1 progeny at those loci (available at http://toxomap.wustl.edu/IIxIII_Typing_Table.html). This result shows that the low reactivity found in some non-archetypal strains that were predicted to react based on the epitope present may instead be explained by the existence of other unique immunodominant epitopes that have yet to be pinpointed.

### Human Sera Peptide ELISA

Although the mouse ELISA results provided promising results, the final objective of *Toxoplasma* serotyping is arguably aimed to assist in human toxoplasmosis. Hence, a total of 34 human serum samples (**Supplemental File 1B**) were used to test the 27 final peptides by ELISA, except the GRA7-II-226 peptide that could not be used in this assay due to insufficient amounts. All the samples reacted to the rSAG1 control antigen, indicating that, although titers varied, antibody levels were high enough to be able to compare reactivity against different polymorphic peptides (**Supplemental File 4**). In addition, all samples from France (FR-X) (de-la-Torre et al., 2013) showed a rSAG1 reactivity matching that from the IgG titers reported based on the ARCHITECT IgG assay (**Supplemental File 1B**, **Supplemental File 4**). The “duplicated” 6I/III, 6II and 7II control peptides (Kong et al., 2003) proved to be reactive against most of their respective serum type samples in our analysis, confirming the validity of our results. Nevertheless, when compared to our GRA6-I/III-213, GRA6-II-214 and GR7-II-214 peptides (which share the same sequence), although values were in general very close to each other, some samples only reacted above the cut-off for one of the duplicates. This indicates that individual variation occurs within each sample and peptide, including peptides from different batches, which highlights the importance of using a large number of peptides to minimize this effect. To make our results more uniform, only our recent batch of peptides was considered for further analysis. All the peptides except GRA5-II-38 elicited a response above the cut-off for at least one of the serum samples. The most reactive peptides were GRA7-II-224, GRA6-I/III-213 and GRA6-II-214, which produced a response above the cut-off limit in 19/34, 15/34 and 12/34 serum samples, respectively. On the other hand, a number of peptides reacted to only a few of the samples: GRA5-I-38, GRA6-II-215 and GRA15-II-477 to only 4/34 samples, and GRA3-II-43, ROP8-I/III-61, ROP8-I/III-305, ROP20-I-331 and hp-I/II-47 to 5/34 samples (**Supplemental File 4**).

From the 34 human serum samples, all but 7 (FR-96, FR-25, FR-27, FR-30, FR-54, FR-58 and JOS1) reacted to at least one of the peptides. It is worth mentioning that some of the samples considered non-reactive (NR) by previous analysis did react, although weakly, to some of the peptides tested in this study: FR-10, FR-19, FR-31, FR-46, FR-48 and FR-49. On the other hand, FR-96 that reacted weakly to the Kong panel of GRA6 and GRA7 peptides did not react to any of the peptides tested in our study (**Supplemental File 4**). Since all these values were very close to their respective threshold, it is possible that slight variations in reactivity between assays may render them positive or negative to specific peptides. Therefore, low values should be carefully interpreted and not be considered individually. Regardless, samples with a strong positive reaction to *Toxoplasma* lysate or rSAG1 antigen but very low, or absent, reaction against multiple strain-specific peptides have been considered in the past as an “atypical” (i.e. non-type 1, 2 or 3) profile (Shobab et al., 2013). Furthermore, 5 samples reacted above the cut-off limit against the majority of the peptides: FR84 (23/26), JOBE (22/26), 9M (21/26), FR72 (20/26) and 279 (19/26). Because they reacted against peptides from different strain types, it is possible that the strain causing the infection was different from the archetypal 1, 2, or 3 strains, or that these patients had a mixed infection. Moreover, these 5 samples, together with NZJ, were the only ones reacting to the PA-14 peptide derived from the TGME49_258400 protein (CCp5A, sporozoite-specific), which was described to be able to distinguish oocyst *vs.* cyst derived infections (Santana et al., 2015).

### A Prediction of the Infecting Strain Can Be Made by Using Peptide Ratios

**Figure 4:**
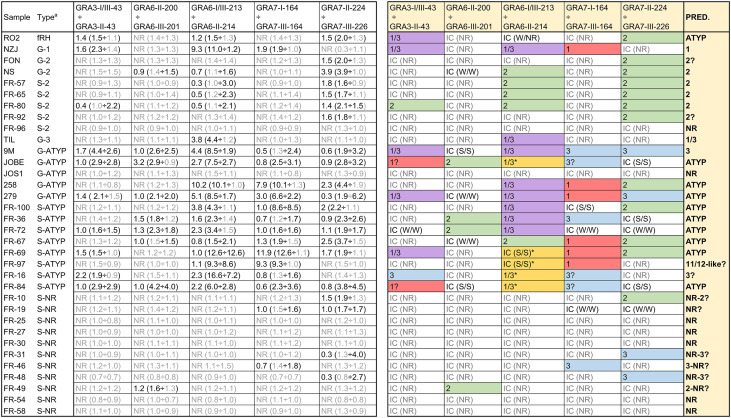
Strain type prediction using human sera reactivity against a panel of *Toxoplasma* polymorphic peptides. On the left, ratios were calculated by dividing individual values from each peptide (in parenthesis). Readers are referred to **Supplemental File 4** for further details on individual values. ^a^ G and S indicate the type of analysis (genotyping and serotyping, respectively) used to detect the strain type. ATYP: Atypical, non-archetypal (non-type 1, 2 or 3). NR: non-reactive. On the right, a prediction was made based on the ratio for each specific peptide couple and the strain causing the infection inferred considering all individual predictions. IC stands for inconclusive. For IC samples, the strength of the reactivity against each peptide is shown in parenthesis as NR (non-reactive), W/W (weak/weak) or S/S (strong/strong). * denotes an unusual strong reactivity to both peptides in the GRA6-I/III-213 *vs.* GRA6-II-214 ratio.

As described above for mice, a ratio was calculated by dividing individual values from the most reactive peptides by their homologous counterpart versions. While the GRA5-I-38 *vs.* GRA5-II-38 ratio proved useful with murine samples, that was not the case with human sera: GRA5-II-38 did not react to any of the samples and GRA5-I-38 only reacted weakly to very few sera (**Supplemental File 4**). On the other hand, two new ratios were used here compared to mice samples: GRA6-II-200 *vs.* GRA6-III-201 and GRA7-I-164 *vs.* GRA7-III-164. In addition, the GRA7-II-224 peptide was compared to GRA7-III-226 instead of GRA7-III-224 on account of its stronger reactivity. Despite these differences and considering each one of the peptide combinations as a whole, a prediction of the strain that caused the infection for each sample was performed (**Figure 4**). Predictions from all but 2 samples matched the information gathered in the past by genotyping or serotyping techniques. Of note, the strains causing the infection in the human patients included in this study were in most cases not isolated and thus the actual initially suspected strain may not be accurate or even a mixture of strains be present, which renders the analysis more difficult.

R02 sera was derived from a patient known to be accidentally infected with the mouse virulent fRH (f=French) strain, which is not RH nor a type 1 strain. Its reactivity pattern with the diagnostic peptides (only reacted weakly to some peptides from different types) supports that designation, that it is atypical, neither type 1, 2 or 3. Another sample, 279, is non-archetypal, but possesses the GRA6 I/III-213 epitope, and reacted positively with that peptide, despite the fact that it possesses a novel GRA6 allele. Further, it has a type 1 allele at GRA7, and as expected, did not react with either the GRA7 II-224 or GRA7 III-226 peptide. Finally, sera from JOS1 also failed to react with the GRA6 (I/III and II) and GRA7 (II and III) peptides, an expected result since it possesses a novel epitope at GRA6 and a type 1 epitope at GRA7. These data support the notion that the reactivity profile at the GRA6 and GRA7 antigens is epitope specific.

We also included in our study several samples previously serotyped as NR (all French samples) to test if an expanded set of peptides would be able to identify their type. Although some NR samples showed a weak reactivity against few peptides, most of them failed to react to any peptide, confirming previous results (de-la-Torre et al., 2013). The reason underlying this lack of reactivity to different peptides despite keeping a strong reactivity against rSAG1 is currently unknown. Finally, 5 samples (JOBE, FR-16, FR-69, FR-84 and FR-97) reacted strongly to both GRA6-I/III-213 and GRA6-II-214, although being higher with the former in all but FR-69, which showed similar reactivity with both peptides (**Figure 4**, **Supplemental File 4**). Although this unusual pattern is similar to the one described above for the Cougar (HG11) mouse sample, these human samples reacted strongly to several other peptides (including PA14 for JOBE and FR-84), which indicates the infection was likely produced by an atypical strain or that the patients had a mixed infection. Nevertheless, among these samples, FR-97 displayed a profile that is similar to that observed for the Cougar-infected mice. As opposed to the other four samples, besides the strong reaction against both GRA6 peptides it only reacted to the GRA7-I-164 peptide (which shares the amino acid sequence with Cougar and HG12 Ari and Ray strains), while failing to react to the GRA7-224 and 226 peptides (**Supplemental File 4**). To the best of our knowledge, the Cougar and HG12 strains have not been isolated in France; however, it is possible that this patient acquired the infection abroad or that the spectrum of strains present in France and other European countries are not yet fully characterized.

### HG11/12 Strains Can Be Resolved by Their Distinct Reactivity Against GRA6-I/III-213 and GRA6-II-214

Because the C-terminal GRA6 amino acid sequence is identical in the HG11 Cougar and HG12 Ari and Ray strains (**Figure 2**), we further sought to investigate whether the unique GRA6-I/III-213 *vs.* GRA6-II-214 pattern observed both for the Cougar infected mice sample and some human samples could be a signature of HG11/12 infections. For this, we tested the two GRA6 peptides (I/III-213 and II-214) against a new panel of sera from CD1 mice infected with different HG12 isolates (**Table 2**). Mouse sera were collected at 1 month post-infection, and since these strains are highly virulent to outbred CD1 mice, animals needed to be treated with sulfadiazine (0.4 mg/ml in drinking water) at the time of infection or upon detection of clinical signs to survive acute infection. All HG12 infected mice developed a strong mixed reactivity against GRA6-I/III-213 and GRA6-II-214, but with a predominant GRA6-I/III-213 response, which correlates with the results described above for the Cougar strain. Moreover, all these samples also failed to react to the GRA7-II-224 peptide, which is in accordance with the type 1 sequence for this peptide in Cougar, Ari and Ray strains (**Figure 2** and **Table 2**). Taken together, these results support the notion that HG11/12 strain samples elicit a unique strong reaction against both GRA6-I/III-213 and GRA6-II-214 peptides combined with the absence of reactivity against GRA7-II-224.

**Table 2:** HG12 mouse serum samples react distinctively against GRA6 peptides.

Mouse ID	Dose^a^	Strain^b^	Genotype	Sulf.^c^	rSAG1	GRA6-I/III-213÷GRA6-II-214	GRA7-II-224
4338	NA	UI		Y	0.7		
4336	NA	UI		Y	0.7		
4334	1,000	3045	HG12	Y	14.4	1.0 (2.0÷2.1)	0.9
4328	1,000	3133	HG12	Y	16.3	4 (7.2÷1.8)	1.1
4329	1,000	3133	HG12	Y	17.4	1.9 (5.9÷3.1)	1.1
4332	1,000	3133	HG12	Y	11.5	2.2 (8.4÷3.8)	1.4
4331	1,000	3142	HG12	Y	18.6	3.3 (15.7÷4.8)	1
3916	50	3429	HG12	N	24.0	1.6 (5.8÷3.6)	0.7
3917	50	3429	HG12	N	23.5	1.4 (13.5÷9.4)	1.2
BC-3	50	3675	TYPE II	N	23.7	0.1 (1.1÷13.7)	12.2

Mouse sera were collected at 1 month post-infection. GRA6-I/III-213 vs. GRA6-II-214 ratio was calculated by dividing individual normalized values from each peptide (in parenthesis). Threshold values below which each sample-peptide reaction is considered negative are greyed out. Green highlighting represents positive rSAG1 values, while orange and blue colors represent GRA6-I/III-213 vs. GRA6-II-214 ratios above (or close to) and below 1, respectively. (a) Number of tachyzoites unless otherwise stated. NA: not available. (b) UI: uninfected. (c) Sulf.: sulfadiazine treatment (0.4 mg/ml in drinking water) was applied at the time of or early after infection to avoid mortality in the acute phase.

### Different Allele Combinations Are Formed When Comparing the Amino Acid Sequence of GRA3, GRA5, GRA6, and GRA7 Peptides

**Figure 5:**
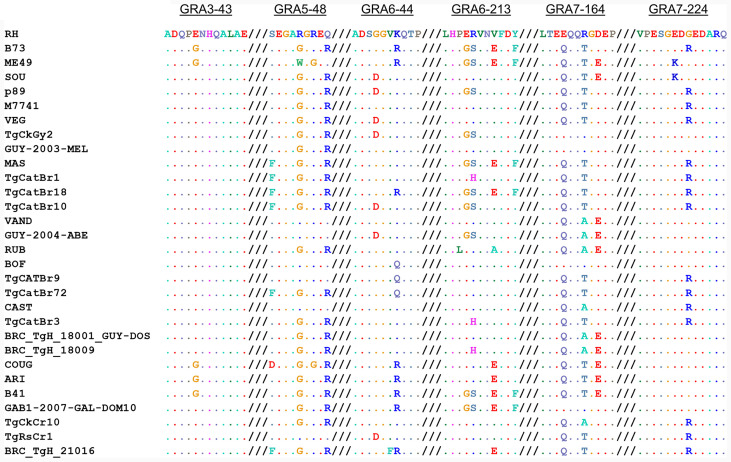
Allele combinations in GRA peptide amino acid sequences among 64 *Toxoplasma* strains. Amino acid sequence alignment from GRA3-43, GRA5-38, GRA6-44, GRA6-213, GRA7-164 and GRA7-224 peptides (from left to right, respectively) in the 64 strains available on ToxoDB shows the possible combinations that give rise to different allele groups. The different possible combinations are aligned and a representative strain from each group is given, where dots indicate identical amino acids. For further details and individual sequences for each strain we refer readers to **Supplemental File 5**.

When we aligned the amino acid sequences of the GRA peptides used in the ratio analysis and compared the 64 strains available on ToxoDB, we observed that the possible combinations give rise to different allele groups. Namely, 2, 5, 5, 6, 5, and 3 different combinations were observed for GRA3-43, GRA5-38, GRA6-44, GRA6-213, GRA7-164, and GRA7-224 peptides, respectively (**Supplemental File 5**). Furthermore, 30 different allele groups were detected when all these peptides were compared together (**Figure 5**). Among these, all the 6 type 1 strains, together with the type 7 TgCkBr141 strain, had the same peptide sequence combination. In addition, 4 of the type 6 strains, including BOF and FOU, had an almost identical combination compared to the type 1 strains, except one amino acid in the GRA6-44 peptide. On the other hand, the type 2 and 3 strains showed different allele combinations and were distributed in 3 and 5 different groups, respectively. Besides the type 1 group, the next larger group was formed by 5 strains from the types 3, 8, 9 and 16, such as G662M, P89 or CASTELLS. By contrast, most of the groups were only composed of one or two strains. Despite this, it is worth mentioning that numerous combinations were very similar except for one of the peptides and sometimes differing in only one amino acid; therefore, they could be considered virtually identical in practice (**Figure 5**, **Supplemental File 5**). Taken together, these results show that different strains can react in a similar way to these peptides on account of their sequence similarity, although it is possible that the presence of particular immunodominant regions may differ within strains. Therefore, although epitope is predictive in archetypal strains and some non-archetypal strains, it is the reactivity pattern across multiple peptide pairs that should be used to define a specific signature or fingerprint for each strain.

## Discussion

Serological typing relies on the identification of long-lasting antibodies produced against parasite proteins that are polymorphic among distinct strains (Kong et al., 2003; Garcia-Ispierto et al., 2010; Vaudaux et al., 2010; Herrmann et al., 2012; McLeod et al., 2012; Hutson et al., 2015). Through a large-scale peptide array assay we recently identified a large number of novel *Toxoplasma* antigenic peptides able to discriminate between infections caused by different strain types (Arranz-Solís et al., 2019). However, arrays are expensive and not always available or transferable. Hence, in the present work we sought to further investigate the potential diagnostic properties of the most promising peptides by means of a more accessible and affordable ELISA test with a larger collection of murine and human serum samples. To that end, a total of 28 *Toxoplasma* allele-specific peptides mainly from dense granule (GRA3, GRA5, GRA6, GRA7 and GRA15) and rhoptry (ROP8 and ROP20) proteins were designed and synthetized.

Our results show a number of peptides that discriminate between mice infected with different type 1, 2 and 3 strains. Among these, GRA6-I/III-213, GRA6-II-214, GRA7-II-224 and GRA7-III-224 were already described (Kong et al., 2003), whereas GRA3-I-38, GRA3-II-38, GRA5-I/III-43, GRA5-II-43, GRA7-II-226, GRA7-III-226 and GRA15-II-477 are novel peptides. In accordance with earlier studies, we detected a high degree of individual variability for each peptide and sample (Sousa et al., 2008; Maksimov et al., 2012b). In an attempt to significantly improve *Toxoplasma* serotyping, we calculated a ratio between RIPC values of different strain versions of the same peptides. A similar comparison between GRA6 and GRA7 I/III and II peptides was previously made by McLeod et al. (2012). In that work, when a sample reacted strongly to both peptides, different designations were given depending on the predominance to one of the two peptides. Similarly, we have used a numeric ratio with GRA3, GRA5, GRA6 and GRA7 peptide pairs to determine which one is predominant and hence predict the strain type regardless of the reactions that happen in low-specific or low-sensitive peptides. A clear example of this is the combination of GRA5-I-38 and GRA5-II-38: if considered individually, some samples are difficult to allocate into one group, as they react weakly to only one of them or strongly to both. However, when the ratio is used, type 1 (>1) and type 2 (<1) strains can readily be differentiated. Unfortunately, all our attempts to resuspend the GRA5-III-38 peptide were unsuccessful. Had it worked, this peptide would have also allowed us to individually differentiate type 3 from 1 and 2 strains. Peptides able to distinguish type 2 *vs.* non-type 2 (Kong et al., 2003), type 1 *vs.* type 3 (Xiao et al., 2009) and type 2 *vs.* type 3 infections (Maksimov et al., 2012a) have been previously described. In addition to these, we show peptide combinations able to differentiate type 3 *vs.* non-type 3 (GRA3), 1 *vs.* 2 (GRA5) and 2 *vs.* 3 (GRA7) strains. Nevertheless, it is worth mentioning that only one type 1 mouse infection was available in our study, and given the individual variability shown by other samples, further investigation is needed with a larger number of type 1 serum samples to confirm these results. Be that as it may, we confirmed the utility of these GRA peptides by means of a proof of concept where we accurately determined the allele type for each gene using serum samples from mice infected with F1 progeny derived from a type 2×3 cross (Sibley et al., 1992).

In addition to the variability in the peptide recognition among individuals infected with the same strain, a recent report showed that chickens and turkeys experimentally infected had a peptide recognition pattern that changed over time (Maksimov et al., 2018). Herein, we compared samples from mice infected with the type 2 PRU and type 3 CEP strains under a wide variety of experimental conditions: inoculum dose, parasite stage, route of infection, post-infection treatment, time after infection and mouse breed. As expected, differences in the peptide recognition were observed within the same strain. However, since there was a high number of different possible combinations of parameters and few individual samples, comparison between animals could not be made. Regardless, this adds further evidence as to the high individual variability even in the same group of animals reported previously by others (Sousa et al., 2009; Maksimov et al., 2013; Maksimov et al., 2018). In our study, however, a number of peptides showed a constant strain-specific reactivity irrespective of the experimental variables: GRA3-I/III-43 and GRA6-I/III-213 in type 3 and GRA5-II-38, GRA6-II-214 and GRA7-II-224 in type 2 strains reacted in all cases in a frequently strong manner.

When we tested the peptides with our panel of human samples, we observed different levels of reactivity compared to that of murine samples. While some peptides that reacted strongly in mice were very weak in human samples, other peptides that did not elicit any response with any mouse serum were able to react to some human samples. Nonetheless, it is important to remark that in our study most of the human samples were either genotyped or serotyped as type 2 or atypical (non-type 1/2/3) strains, while only one type 3 sample was included. Despite this shortcoming, with the current panel of peptides we were able to correctly predict the *Toxoplasma* type in the great majority of human samples. This further supports the notion that even though some peptides may react differently depending on the host and individuals, a prediction can be made if a large number of peptides is used (Arranz-Solís et al., 2019).

Although we and others have shown that a distinction between the archetypal strains (type 1, 2 and 3) is possible by serological typing, non-archetypal strains are harder to investigate due to the lack of specific peptides. Maksimov and colleagues attempted to distinguish cats infected with non-type 1/2/3 strains, but they observed a high reactivity against type 2 and, specially, type 1/3 peptides (Maksimov et al., 2013). The current availability in ToxoDB of the whole genome sequences of 64 different *Toxoplasma* strains, including non-archetypal ones (Lorenzi et al., 2016), makes it possible to start investigating specific allelic variations between these and 1/2/3 strains. Unsurprisingly, non-archetypal strains share, in most cases, the sequence of one of 1, 2 or 3 types in different combinations. For example, type 7 CAST shares the sequence of type 1/3 in GRA3-43 peptides, type 1 in GRA5-38, type 1/3 in GRA6-213/214 and type 3 in GRA7-224/226. Another important hindrance in the advancement of non-archetypal strains serotyping is the scarcity of validated serum samples from animals or patients infected with these strains. Herein, we used sera from mice experimentally infected with a number of different atypical strains (type 4 MAS, type 6 BOF, type 7 CAST, type 9 P89, type 10 GUY-DOS and type 11 Cougar). In agreement with what was reported by Maksimov et al. (2013) and the performed alignments, we observed a high reactivity of these samples to few peptides from type 1/3 (GRA3-I/III-43 and GRA6-I/III-213) and/or type 2 (GRA6-II-214 and 215) strains, which correlated in most cases with the specific sequence shared with their respective type. Similarly, Kong and colleagues observed a type 1/3 profile with the GRA6-I/III-213 peptide in mice and human CAST samples that were believed to belong to the type 1 group (Kong et al., 2003), and which is currently classified as type 7 (Lorenzi et al., 2016). As suggested by others and confirmed here, non-archetypal strains contain unique combinations of alleles at any given genetic locus, highlighting the need for the inclusion of a considerably larger number of peptides from different genes to avoid a misprediction of the strain type. This feature should be further exploited in future experiments by employing new peptides from polymorphic antigens that have dissimilar allele segregation not only compared to the archetypal types but also within non-archetypal strains.

In our study, when the ratio between the GRA6-I/III-213 and GRA6-II-214 peptides was analyzed for the type 11 Cougar mouse serum sample, a distinct pattern was observed: while this ratio showed a clear predominance of the respective type-specific peptide in type 1, 2, and 3 strains, it reacted against both 2 and 1/3 peptides, but stronger to the latter, in the Cougar strain. Likewise, a similar pattern with these two GRA6 peptides was observed in mice infected with different recombinant type 12 strains. Considering that the amino acid sequence in the C-terminal epitope of GRA6 is identical in the type 11 Cougar and type 12 Ari and Ray strains, this could be considered as a “HG11/12” profile. Together with the aforementioned mixed GRA6 reactivity, a further trait of this profile would be an absence of reactivity to the GRA7-II-224 peptide, as Cougar, Ari and Ray have the type 1 version for this peptide. In line with this, a mixed reactivity against GRA6 type 1/3 and 2 peptides but not GRA7 type 2 peptides has been already reported and shown to be a characteristic feature of the type 12 Ari and Ray strains (McLeod et al., 2012). Indeed, McLeod et al. described a similar reactivity of type 12 samples to both GRA6 peptides and defined a “II=I/III” profile. Although in our study some non-archetypal human samples showed a similar pattern, most of them had a close but stronger reactivity against GRA6-I/III-213 compared to GRA6-II-214, a profile designated by McLeod as “I/IIIa”. Whether this is something specific of the Ari or Ray strains compared to other type 11 or 12 strains deserves further investigation. In fact, the B41 strain, currently classified within haplogroup 12 together with Ari and Ray (Lorenzi et al., 2016), does not share the same sequence in that epitope of GRA6, but rather it has a type 2 sequence. This correlates with the fact that B41 has been described to be an F1 progeny from a type 2 and Ray/WTD3 (belonging to haplogroup 12) cross (Minot et al., 2012). Thus, although serum samples from patients or animals infected with this B41 strain were not tested, it is tempting to hypothesize that the reactivity against the GRA6 peptides could be different from the “HG11/12” of Cougar, Ari or Ray, but similar to PRU or ME49 strains. Nevertheless, it is also possible that other non-archetypal strains may elicit this GRA6 reactivity or that this particular pattern may be observed when a mixed infection is present, as most of the patients showing this HG11/12 profile for GRA6 in our study did also react strongly to several other peptides. Hence, further samples from animals and patients infected with different strains need to be tested to investigate whether this strong and near equal (or slightly higher) reactivity against GRA6-I/III-213 compared GRA6-II-214 is a specific signature of HG11/12 strains. This might prove extremely useful in North America, where type 2, 12 and 3 account for most of strains isolated from wild-life, with the type 12 commonly being found in severe cases of ocular and congenital toxoplasmosis (Grigg et al., 2001; Khan et al., 2011; Minot et al., 2012; Schumacher et al., 2020). In addition, the Cougar strain has been epidemiologically linked to a major waterborne outbreak occurred in Victoria, British Columbia, Canada (Aramini et al., 1998; Aramini et al., 1999), and recently an outbreak in Wisconsin, USA, was reported to be caused by type 12 strains from contaminated venison (Schumacher et al., 2020).

Apart from the “HG11/12” profile, we observed two other characteristic patterns in the non-archetypal serum samples. Firstly, despite having similar high reactivity to the *Toxoplasma* lysate or rSAG1 antigen, some samples reacted to none, or weakly to few, of the peptides. This was apparent in both mouse and human samples, although only few of the human samples failed to react to any of the peptides. This profile, termed non-reactive, has been previously described even after using a high number of peptides, and has been associated with infections by atypical strains, especially in ocular toxoplasmosis patients (Kong et al., 2003; Maksimov et al., 2012a; Maksimov et al., 2012b; Shobab et al., 2013). In these studies, the lack of reactivity against type 1, 2 and 3 strain-specific peptides by samples from non-archetypal strain infections was attributed to possible differences in the amino acid sequence. However, with the current availability of the whole genome sequences of several non-type 1/2/3 strains (Lorenzi et al., 2016), we have observed that the absence of reactivity often does not correlate with differences in amino acid sequence, as even strains sharing the sequence with type 1, 2 and/or 3 strains do not react to their respective peptide. Instead, this non-reactive profile may be explained by the presence of other immunogenic epitopes in the proteins of those strains that are dominant and prevent other epitopes, such as the ones from the peptides, from reacting (Arranz-Solís et al., 2019). Alternatively, it could be possible that this lack of reactivity against short strain-specific peptides may be caused by the Hoskins paradox, aka original antigenic sin. This term was coined more than 60 years ago after observations made in patients that showed a reduced vaccine effectiveness against influenza virus (Thomas Francis, 1960; Monto et al., 2017). The prior exposure to a particular strain of the virus produced a diversion of the antibody response against a second infecting strain to focus on cross-reactive immunodominant epitopes. Although this phenomenon has not been described in *Toxoplasma* and is out of the scope of the present work, superinfection with a different strain than the original strain could provide a plausible explanation for the lack of reactivity which is only observed in human serum samples.

Regardless, the second pattern frequently observed in non-1/2/3 type serum samples was a strong reaction against the majority of the peptides, which was observed exclusively in human samples. This could represent a specific pattern for certain non-archetypal strains, although since we did not observe this pattern in mice experimentally infected with several of them, the possibility that this pattern may just have been caused by mixed natural infections cannot be ruled out (Aspinall et al., 2003; Nowakowska et al., 2006; Vaudaux et al., 2010). Interestingly, together with NZJ, these samples were the only ones reacting to the PA-14 peptide derived from the sporozoite-specific protein CCp5A. This protein has been shown to be able to differentiate infections caused by oocysts (Santana et al., 2015). Therefore, although the origin of infection in the patients included in our study is unknown, it is possible that the reactivity against PA-14 was either the result of infection by ingestion of oocysts, or caused by the high levels of antibodies found in these patients.

Finally, apart from the low type-specificity of particular peptides that may be explained by a strong immunoreactivity in the non-polymorphic parts of these peptides, identical sequences in the peptides employed did not always match with the observed reactivity in different strains. For instance, although several strain types share the same amino acid sequence, only serum samples from mice infected with type 3 strains and type 7 CAST were recognized by GRA3-I/III-43 (ADQPENHQALAE). Similar to the non-reactive pattern discussed above, this effect may be accounted for by the presence of other immunogenic epitopes in the protein for certain strains that prevent short peptides from reacting. On the other hand, we have confirmed the importance that single amino acid substitutions may have on the peptide recognition. For example in the GRA6 C-terminal epitope the GS/ER polymorphisms are not important, while the EFDF/VFDY portion is much more relevant (Kong et al., 2003; Arranz-Solís et al., 2019). Similarly, the K/E in position 229 in GRA7-224 and 226 peptides proved to be the most important amino acid. These results, together with the lower reactivity of shorter peptides (for example GRA6-II-214 *vs.* GRA6-II-215) are in accordance with the notion that the polymorphic site and length of peptides play a pivotal role in serological typing (Kong et al., 2003; Arranz-Solís et al., 2019).

In summary, we present novel antigenic peptides able to differentiate type 1, 2 and 3, as well as some non-archetypal strains, by comparing the reaction to different variants of polymorphic regions rather than relying on individual peptides. As a proof of concept, we show that GRA3, GRA5, GRA6, and GRA7 peptides are able to correctly predict the type for each locus in mice infected with strains derived from a type 2×3 cross. This same principle could be applied in future studies to investigate the strain an animal or a patient is infected with. In addition, we also observed reaction patterns that were exclusive of some atypical strains, especially in human samples. This allowed us not only to clearly differentiate them from the three canonical lineages, but also to identify specific profiles from certain groups of strains, such as the HG11/12, which may spur further investigations on these strains and their frequent association with severe toxoplasmosis cases. In order to advance in *Toxoplasma* serotyping, a wider panel of well characterized samples would prove critical to better characterize their reaction against a large pool of polymorphic peptides from different loci described by us and others. This can further advance in the definition of a clear signature for each host and strain, with a particular growing interest in the understudied non-archetypal strains.

## Data Availability Statement

The original contributions presented in the study are included in the article/**Supplementary Material**. Further inquiries can be directed to the corresponding author.

## Ethics Statement

The studies involving human participants were reviewed and approved by Human samples were used according to the Committee on the Use of Humans as Experimental Subjects (COUHES) application No. 0808002869. Written informed consent for participation was not required for this study in accordance with the national legislation and the institutional requirements. The animal study was reviewed and approved by The MIT Committee on Animal Care (assurance number A-3125-01), the Institutional Animal Care and Use Committee (IACUC) at the University of California, Davis (assurance number A-3433-01), and the Animal Care and Use Committee (ACUC) of the Intramural Research Program of NIAID (Animal study protocol; LPD22E) approved all protocols, and all efforts were made to minimize unnecessary distress to the animals. The animal study protocol LPD22E was reviewed and approved by the Animal Care and Use Committee of the Intramural Research Program of the National Institute of Allergy and Infectious Diseases (NIAID) at the National Institutes of Health.

## Author Contributions

Conceptualization, methodology, validation, and formal analysis: DA-S, MG, and JS. Investigation: DA-S, CC, EZ, and MG. Resources: MG and JS. Data curation: DA-S and MG. Writing original draft: DA-S. Writing review and editing: DA-S, MG, and JS. Supervision, project administration, and funding acquisition: JS. All authors contributed to the article and approved the submitted version.

## Funding

Financial support was provided by National Institutes of Health (NIH) grant 1R21EY024593. This work was also supported in part by the Intramural Research Program of the National Institute of Allergy and Infectious Diseases (NIAID) at the NIH grant number AI001017 and by the CNPq and FAEPA in Brazil.

## Conflict of Interest

The authors declare that the research was conducted in the absence of any commercial or financial relationships that could be construed as a potential conflict of interest.

## Supplementary Material

Supplemental File 1 Mouse **(A)** and human **(B)** serum samples used for ELISAs.

Supplemental File 2 Raw mouse ELISA OD values and normalization process.

Supplemental File 3 Individual RIPC and ratio values of antigenic peptides tested with mouse sera by ELISA.

Supplemental File 4 Normalized values of human peptide ELISA.

Supplemental File 5 Amino acid sequence alignment from GRA3-43, GRA5-38, GRA6-44, GRA6-213, GRA7-164 and GRA7-224 peptides in the 64 strains available on ToxoDB shows the possible combinations that give rise to different allele groups.
